# Investigation of Fracture Behavior and Mechanism in High-Speed Precise Shearing for Metal Bars with Prefabricated Fracture-Start Kerfs

**DOI:** 10.3390/ma13184073

**Published:** 2020-09-14

**Authors:** Yuanzhe Dong, Jinqiang Ning, Peng Dong, Yujian Ren, Shengdun Zhao

**Affiliations:** 1 School of Mechanical Engineering, Xi’an Jiaotong University, Xi’an 710049, China; hpd611@163.com (P.D.); renyujian@stu.xjtu.edu.cn (Y.R.); 2 George W. Woodruff School of Mechanical Engineering, Georgia Institute of Technology, Atlanta, GA 30332, USA; jinqiangning@gatech.edu

**Keywords:** metal bar, precise shearing, high-speed, kerf

## Abstract

A laser-assisted high-speed shearing (LAHSS) method has been proposed for metal bars, which prefabricates equally spaced fracture-start kerfs by Nd:Yag laser to make stress concentration, and applies a high-speed load to complete fracture separation. Comparative tests were conducted for Q235, 40Cr, and 304 steel bars, and the effects of fracture-start kerfs and axial clearance were investigated on the fracture section. Moreover, the fracture behavior was demonstrated by numerical simulation, and the micro-fracture mechanism was revealed by fractographic analysis. The numerical simulation results show that the material damage concentrates along with the kerf tips with peak equivalent plastic strain, and the corresponding stress triaxiality drops to almost zero at the kerf tip, which reveals that the material is subjected to pure shearing at kerf tip; the Max. loading force is reduced by 15.2%–29.6%, and the impact energy is decreased by 29.8%–46.9% for the three types of bar material. The experimental results showed that the fracture-start kerfs effectively inhibited the plastic deformation stage, and higher precision blanks were obtained in the LAHSS test: roundness error improved from 2.7%–10.9% to 1.1%–2.6%, Max. bending deflection decreased from 1.3–3.4 mm to 0.4–1.0 mm, and flatness error dropped from 0.9–3.3 mm to 0.3–0.7 mm. The fractographic analysis reveals that the crack initiation is related to alternative V-shape micro-notches at the laser-affected zone; the predominant fracture mechanism involves mode II microvoid coalescence at the main fracture plane; smaller and less elongated dimples were formed in 40Cr steels due to higher number density of grains and pinning effect of second-phase particles compared to Q235 and 304 steel bars.

## 1. Introduction

**Figure 1:**
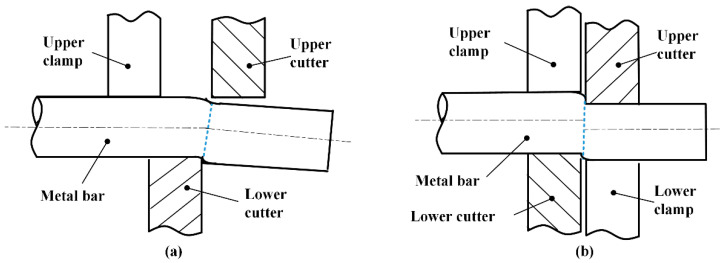
Schematic of different cropping methods. (**a**) Industrial cropping method. (**b**) The high-speed shearing method with radial constraint.

High-quality cropping of blanks from long metal bars is the first process of near-net-shape forming for most mechanical parts. As shown in Figure 1a, the industrial cropping method moves sharp blades downward to cut off blanks at a loading speed below 0.6 m/s. In this process, the bar material is first compressed with a certain degree of bending deflection, and the crack initiation is caused by a progressive accumulation of plastic deformation around the upper and lower blade edges, which not only requires a great enough force especially for high-strength metals, but also deviates the maximum shearing stress plane from the vertical cropping direction. Consequently, the fracture sections are featured by draw-in distortion with enlarged ovality, which usually needs further edge-cutting process.

Several precise cropping methods have been proposed to improve the section quality by changing irregular ductile fracture into controllable brittle or quasi-brittle fracture, mainly including low-load fatigue cropping, pre-shearing cropping, and high-speed shearing. The low-load fatigue cropping method prefabricates V-shape notches and applies a low-frequency cyclic load to complete fracture [1,2]. In this process, fatigue cracks initiate from the notch tips and propagate steadily, which is controlled by loading curves with decreasing frequency [3]. The V-shape notches are processed by cutters with optimal depth as 4–5% of the bar diameter, opening angle of 60–90°, and the minimum tip radius of 0.1–0.2 mm [4]. However, the stress concentration effect is limited by the cutter shape, and the hard material notching is still difficult. Moreover, efficiency is still relatively low (3~6 blanks per minute). The pre-shearing cropping method includes two sub-stages: the bar is pre-sheared at a low speed less than 50 mm/s, and then cropped at a higher speed about 600 mm/s [5]. This method produces flat and vertical cross-sections, but the roundness error is inferior due to the draw-in deformation at the first stage and is unsatisfactory for soft metals such as low-carbon steels.

**Figure 2:**
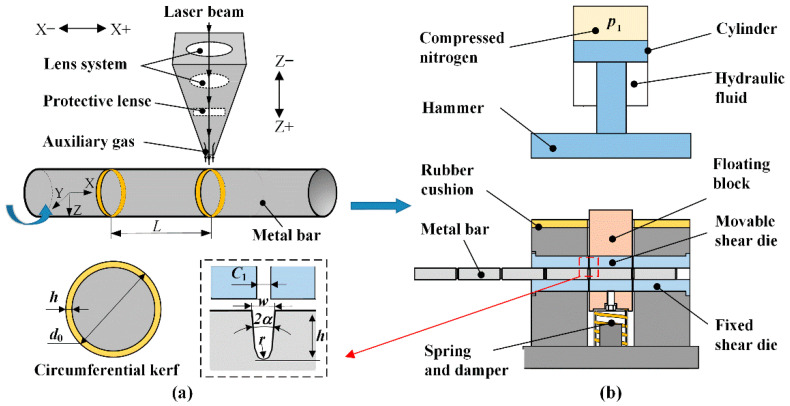
Schematic of the LAHSS method. (**a**) Stage one: fabricate circumferential kerfs by laser. (**b**) Stage two: high-speed shearing of radially constraint metal bar on a power-drop hammer.

High-speed shearing method [6] enhances the brittleness of metals by increasing the loading speed, and the workpiece is usually radially constraint [7], as shown in Figure 1b. Experiments conducted by Chen [8] on an air hammer (about 4.5 m/s) have shown that the section quality can be improved for various kinds of steels and non-ferrous metals, such as aluminum alloys. Song et al. [9] concluded that better cutting quality can be obtained with a loading speed of 5–7 m/s and a temperature of 350–380 °C, as shown in Figure 2b–c. However, the section ovality is still large, and this method still has problems such as large impact load and frequent breakage of blades; the micro-fracture mechanism is not involved in the above research. Recent studies have shown that for the body-centered cubic (BCC) metals, especially mild and medium steels, they represent a considerable range of strain-hardening and sensitivities of fracture toughness to high-speed load [10]. Singh [11] et al. conducted dynamic tensile and compression tests of mild steel at a strain rate of 750 s^−1^, and the results showed that the yield strength increases by 2.5 times, and the ultimate tensile strength increases by 1.18 times. Longère and Dragon [12] applied hat-shape samples into dynamic shear-pressure tests (1000–5000 s^−1^) under low triaxiality, and found that the failure mechanism is determined by the interactions and competition between the void growth ductile fracture and adiabatic shearing. Henschel [13] investigated the Charpy impact tests of hot-rolled 42CrMo and observed the forming of ductile dimples due to nonmetallic inclusions in the crack initiation and unstable crack growth process.

Laser cutting method is especially suitable to process high-precision narrow kerfs (width of 0.1–0.5 mm), and has been applied in cutting or grooving hard-to-cut metal sheets and thin-wall tubes [14]. Laser processing fracture-starting kerf has been industrialized in the quasi-cleavage splitting of the C70S6 engine connecting rod [15]. In this study, a laser-assisted high-speed shearing (LAHSS) method has been proposed, which prefabricates sharp circumferential kerfs on the bar surface by Nd:Yag laser to make stress concentration, and applies a high-speed load to complete separation. Comparative LAHSS tests were conducted for low-carbon Q235 steel with good ductility, medium-carbon 40Cr steel with high strength [16], and 304 stainless steel with good ductility and high fracture toughness ($J_{\mathrm{IC}}$ > 150 kJ/m^2^) [17]. These three types of steel are typically strain-rate sensitive BCC metals, and have been widely used in the machinery and automotive industries in large amounts. FE simulation and micro-fractography analysis are applied to reveal the fracture behavior and micro-fracture mechanism.

## 2. The Laser-Assisted High-Speed Shearing (LAHSS) Method

The working principle of the LAHSS method is illustrated in Figure 2, which includes two sub-stages: fabricate circumferential kerfs by laser, and high-speed shearing on a power-drop hammer. As shown in Figure 2a, a metal bar rotates around the x-axis, and a focused laser beam irradiates onto the bar surface to make local spot melting, vaporization and blown away by high-pressure auxiliary gas; overlapped holes continuously connect into a circumferential kerf, and material in depth is removed layer by layer in multi-pass processing. In sequence, equally spaced circumferential kerfs are processed according to cropping length *L* through the movement of the laser head.

As shown in Figure 2b, the bar is radially constrained in a double-shearing die, and the first two kerfs are placed between the movable shear die and fixed shear die with an axial clearance of $C_{i}$, which should be as small as possible. The nitrogen gas inside the cylinder is initially compressed by hydraulic fluid, and stores a great deal of potential energy with a pressure of $p_{i}$; the movable shear die is fastened inside the floating block, which is replaceable according to the bar diameter. In the impact stroke, the hydraulic fluid is rapidly drained into the oil tank through a valve, so that the hammer is accelerated down under gas pressure and gravity, and strikes the floating block at speed $v_{i}$; two blanks are cropped at a time by the movable shear die throughout the circumferential kerfs. In the return stroke, the hydraulic fluid flows into the cylinder, and the hammer returns to the initial position, so that the floating block rebounds by a spring-damper component.

**Figure 3:**
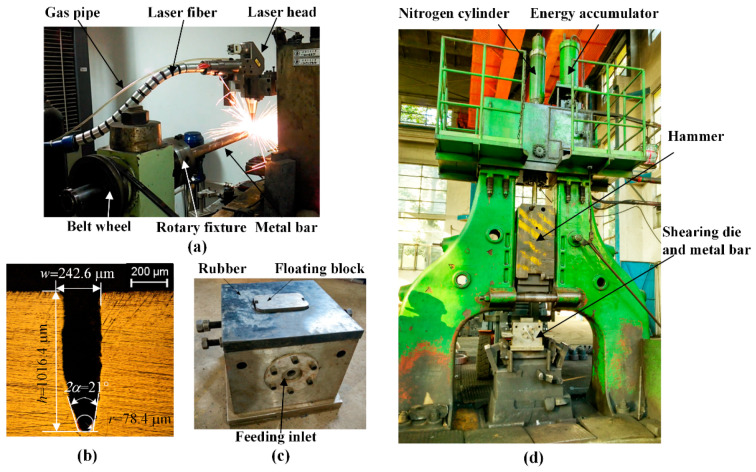
The experimental equipment. (**a**) Laser rotary cutting system. (**b**) Kerf shape for the shearing tests. (**c**) Double-shearing die. (**d**) 35 kJ electro-hydraulic power-drop hammer.

The corresponding LAHSS equipment includes a set of laser rotary cutting system, invented double-shearing die, and a 35 kJ electro-hydraulic power-drop hammer, as shown in Figure 3. The JK300D Nd:Yag laser generator is applied with wavelength of 1.064 μm, beam diameter of 0.2 mm, peak power of 9.6 kW, pulse width of 0.3 ms, and pulse frequency of 50 Hz [14]. The rotary speed was 20 rev/min, and the revolution number was three so that the processing time for each kerf was limited to 9 s. The mass of the hammer *m*_1_ was 1300 kg, and the working stroke was 640 mm after subtracting the height of the shearing die. The total mass *m*_2_ of the floating block and the movable shear die was 28 kg.

The initial kinetic energy E of the hammer is expressed as below, which equals to the sum of work $W_{g}$ by hammer gravity and work $W_{p}$ by gas expansion [18]

(1) $$E=\eta \left(W_{g}+W_{p}\right)=\eta \left(m_{1}gz_{1}+W_{p}\right)=\frac{1}{2}m_{1}v_{1}{}^{2}$$

where $m_{1}$ is the hammer mass, $z_{1}$ is the working stroke; $v_{1}$ is loading speed; *η* is the mechanical efficiency in the impact stroke due to guide friction [19]. Because the time of impact stroke is very short (less than 0.1 s), the gas expansion process is considered as adiabatic in the cylinder. The gas expansion work $W_{p}$ is calculated as

(2) $$W_{p}=\frac{p_{1}V_{1}-p_{2}V_{2}}{k-1}=\frac{1}{k-1}p_{1}V_{1}\left[1-{\left(1-\frac{V_{1}}{V_{2}}\right)}^{k-1}\right]$$

where $V_{1}$ and $V_{2}$, respectively, are the gas volume before and after expansion; $p_{1}$ and $p_{2}$ are the corresponding gas pressures; the gas adiabatic constant *k* is 0.4 for nitrogen.

To obtain complete fracture, the initial kinetic energy *E* should be greater than the fracture energy of the metal bar. This new method introduces fracture-starting kerf to make stress concentration, which not only restrains the plastic deformation of the adjacent material, but also reduces loading force and impact energy. In the scope of line-elastic mechanics, the stress concentration factor can be increased by increasing kerf depth *h*, or decreasing tip radius *r* and opening angle 2*α* [20]. The kerf depth *h* is suggested as about 3.5% of the bar diameter [14].

## 3. Experimental Tests and FE Modeling

### 3.1. Materials and Experimental Tests

**Figure 4:**
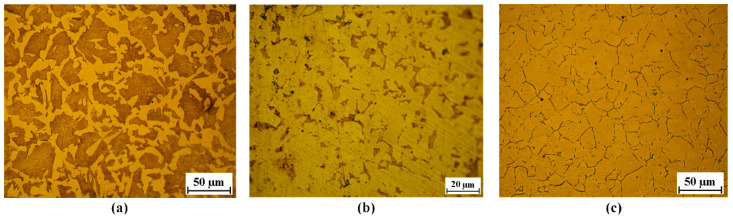
Metallographic structure of bar materials. (**a**) Q235 steel with Vickers hardness of 168.7 HV. (**b**) 40Cr steel with Vickers hardness of 260.1 HV. (**c**) 304 steel with Vickers hardness of 182.3 HV.

Three types of bar material were investigated in this study, Q235 steel (0.2% C), 40Cr steel (0.4% C, 0.9% Cr and 0.7% Mn), and 304 stainless steel (0.06% C, 18.3% Cr, 8.2% Ni and 0.8% Mn). As cropping blanks from long bars is the first process before forging, the commercial supply state of these metal bars is annealed, with a diameter of 26 mm and a total length of 2000 mm. The metallographic structure and average Vickers hardness are shown in Figure 4: the Q235 and 40Cr steels were chemically etched with a mixed solution of nitric acid (4%) and alcohol, and the metallographic structure was mixed with ferrite and pearlite; the 304 steel was processed by electrolytically etched in the 10% oxalic acid solution with an electric current density of 1 A/cm^2^ for 90 s, and the metallographic structure was mainly austenite. The average Vickers hardness was obtained using a Vickers hardness apparatus with a load of 0.5 kg for 15 s.

The kerf shape for the shearing tests is shown in Figure 3b, with depth *h* of 1.0 mm, width *w* of 0.24 mm, bottom opening angle 2*α* of 21°, and tip radius *r* of 0.078 mm. Half the number of bars were preprocessed circumferential kerfs according to cropping length *L* of 100 mm. Comparative shearing tests were conducted for the bars with and without kerfs, and each test was repeated 3 times. Based on Chen [8] and Song’s [9] results, the loading speed *v*_1_ was adjusted to 4.9 m/s with an initial pressure of 1.0 MPa; the initial impact energy was calculated as 15.6 kJ. The motion characteristics of the hammer were recorded by a high-speed camera. The axial clearance was a major factor in the section quality and was adjusted to 0.2 mm, 1.0 mm and 2 mm, respectively; the radial clearance was 0.2 mm. The processing parameters in the cropping tests are listed in Table 1.

### 3.2. FE Modeling of the High-Speed Shearing Process

**Figure 5:**
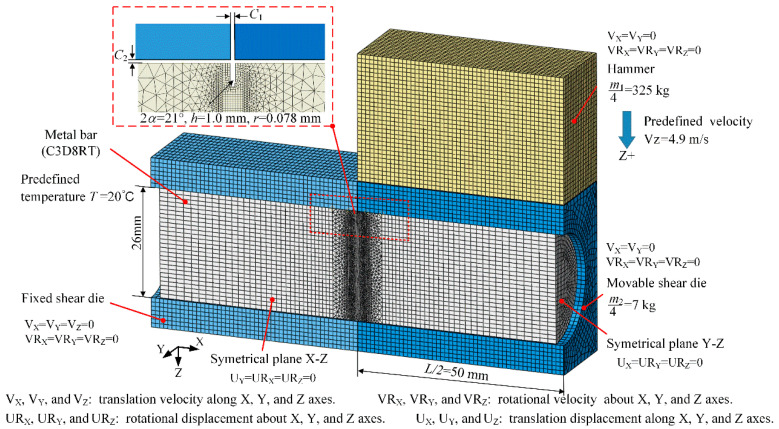
The FE model of the high-speed shearing. V_X_, V_Y_, V_Z_: translation velocity. VR_X_, VR_Y_, VR_Z_: rotational velocity. U_X_, U_Y_, U_Z_: translation displacement. UR_X_, UR_Y_, UR_Z_: rotational displacement.

Figure 5 illustrates a 1/4 symmetry FE model, which is established using Abaqus explicit dynamic modules coupled with thermal-mechanical analysis. The kerf zone and far ends of the bar is established with first-order and reduced integrated hexahedral elements C3D8RT at, which has been widely used to avoid the volumetric locking problem due to second-order elements [21,22], such as C3D20T or C3D20RT elements, for large elastic-plastic deformation and fracture process simulation of almost incompressible metals; tetrahedron C3D4T elements are applied at the transition zones. The kerf and its adjacent zone are meshed with high-level refinement: the minimum element size is 0.037 mm at the kerf tip, and the maximum element size is 1.0 mm at the far end of the bar; the element number of the bar is 1,406,158. The hammer and movable shear dies are established with R3D4 rigid elements. The boundary conditions are as shown in Figure 5; the hammer is applied an initial speed of 4.9 m/s along the z-axis direction. Contact properties are defined as hard contact for normal behavior and penalty friction formulation for tangential behavior; the friction coefficient is set at 0.2 for steels. The axial clearance $C_{1}$ and radial clearance $C_{2}$ are both 0.2 mm considering the actual condition.

The plastic flow stress of the bar materials is described by the Johnson–Cook plastic model.

(3) $$\bar{\sigma }=\left(A+B{\bar{\varepsilon }}_{p}^{n}\right)\left[1+Cln\left(\frac{{\dot{\bar{\varepsilon }}}_{p}}{{\dot{\varepsilon }}_{0}}\right)\right]\left[1-{\left(\frac{T-T_{r}}{T_{m}-T_{r}}\right)}^{m}\right]$$

which defines the relationship between equivalent stress $\bar{\sigma }$ and equivalent plastic strain ${\bar{\varepsilon }}_{p}$ at specific strain rate ${\dot{\bar{\varepsilon }}}_{p}$ and temperature T; ${\dot{\varepsilon }}_{0}$ is the reference strain rate at quasi-static condition, $T_{r}$ is the reference temperature of 20 °C.

The equivalent plastic strain ${\bar{\varepsilon }}_{p}$ is expressed as below [23], which is a measure of the degree of plastic deformation

(4) $$\left\{ \begin{array}{l} {\bar{\varepsilon }}_{p}=\sum {\dot{\bar{\varepsilon }}}_{p}dt\\ {\dot{\bar{\varepsilon }}}_{p}=\sqrt{\frac{2}{9}[{\left({\dot{\varepsilon }}_{1}-{\dot{\varepsilon }}_{2}\right)}^{2}+{\left({\dot{\varepsilon }}_{2}-{\dot{\varepsilon }}_{3}\right)}^{2}+{\left({\dot{\varepsilon }}_{3}-{\dot{\varepsilon }}_{1}\right)}^{2}]} \end{array}\right.$$

where ${\dot{\bar{\varepsilon }}}_{p}$ is the equivalent plastic strain rate; ${\dot{\varepsilon }}_{1}$, ${\dot{\varepsilon }}_{2}$, and ${\dot{\varepsilon }}_{3}$ are the principal strain rates; *dt* is the time increment. The fracture process is defined by the Johnson–Cook cumulative damage model as expressed in Equation (5) [24], where *D* is the cumulative damage value, and fracture is allowed to occur when *D* ≥ 1.

(5) $$\left\{ \begin{array}{l} D=\sum \frac{\Delta {\bar{\varepsilon }}_{p}}{{\bar{\varepsilon }}_{f}}\geq 1\\ {\bar{\varepsilon }}_{f}=\left[d_{1}+d_{2}\exp(d_{3}\sigma *)\right]\left[1+d_{4}\ln\left(\frac{{\dot{\bar{\varepsilon }}}_{p}}{{\dot{\varepsilon }}_{0}}\right)\right]\left[1+d_{5}{\left(\frac{T-T_{r}}{T_{m}-T_{r}}\right)}^{}\right] \end{array}\right.$$

The equivalent fracture strain ${\bar{\varepsilon }}_{f}$ is dependent on stress triaxiality σ*, equivalent plastic strain rate ${\dot{\bar{\varepsilon }}}_{p}$, and temperature T; $\Delta {\bar{\varepsilon }}_{p}$ is the increment of equivalent plastic strain. The material parameters *d*_1_ to *d*_5_ are presented in Table 1. The stress triaxiality is defined as $\sigma *=\sigma_{m}/\bar{\sigma }$:

(6) $$\left\{ \begin{array}{l} \sigma_{m}=\frac{\left(\sigma_{1}+\sigma_{2}+\sigma_{3}\right)}{3}\\ \bar{\sigma }=\sqrt{\frac{1}{2}\left[{\left(\sigma_{1}-\sigma_{2}\right)}^{2}+{\left(\sigma_{2}-\sigma_{3}\right)}^{2}+{\left(\sigma_{1}-\sigma_{3}\right)}^{2}\right]} \end{array}\right.$$

where $\sigma_{1}$, $\sigma_{2}$, and $\sigma_{3}$ are the principal stresses; $\sigma_{m}$ is the average of the three principal stress; $\bar{\sigma }$ is the Von Mises equivalent stress. The σ* reflects the stress state of local material in the fracture process [25,26]: it is mainly subjected to tensile stress for positive σ*, and under compression for negative value; the value of zero reveals that the shear stress plays a major role in the fracture process.

A series of mechanics tests need to be done to obtain the 10 parameters for each type of material. The constitutive parameters *A*, *B*, *C*, *n*, and *m* are obtained by fitting the true stress–plastic strain curves in tensile or compression tests at different temperatures and strain rates. The damage parameters *d*_1_ to *d*_5_ are obtained by fitting the fracture strains in tensile, compression, shearing (or torsion) tests, at different stress triaxiality, strain rate, and temperature. The Q235, 40Cr, and 304 are commonly used industrial steels, and a lot of tests have been done on these materials. Appropriate material parameters *A*, *B*, *C*, *n,* and *m* are cited in Table 2; as cropping of blanks is the first process before forging, the annealed bar material has not yet undergone hardening and tempering.

## 4. Results and Discussion

### 4.1. Fracture Behavior Analysis

**Figure 6:**
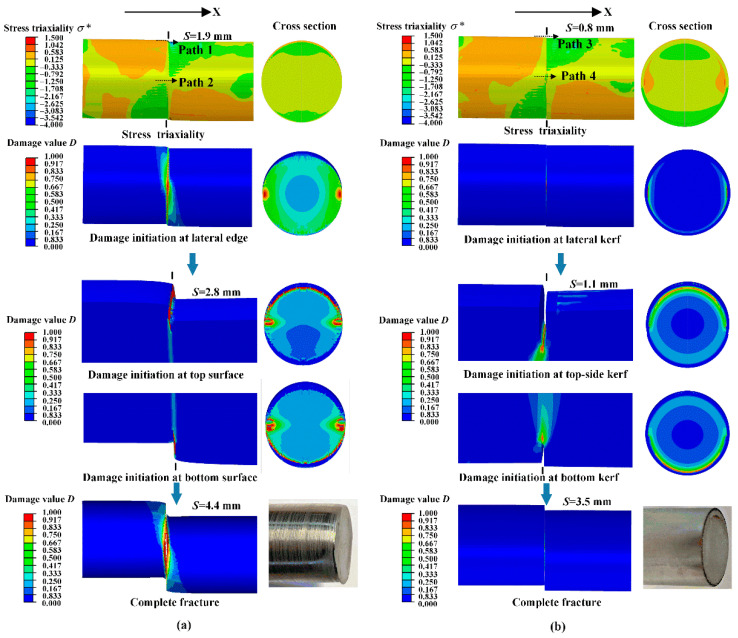
Comparison of fracture behavior of 40Cr bars with and without circumferential kerf, by stress triaxiality σ*, and cumulative damage value *D*. (**a**) Initial kerf depth *h* = 0 mm. (**b**) Initial kerf depth *h* = 1 mm.

The stress triaxiality σ*, cumulative damage value *D*, and equivalent plastic strain ${\bar{\varepsilon }}_{p}$, are analyzed to reveal the fracture behavior in the shearing process, which have been expressed in Equations (4)–(6). Taking the 40Cr bar as an example, for kerf depth *h* of 0 mm, the damage initiates around the edges of upper and lower blades at shearing displacement *S* of 1.9 mm (Figure 6a). At *S* of 2.8 mm, crack seams form at the upper and lower surface, and the plastic deformation deviates the maximum shearing stress plane from the vertical direction. Consequently, inward-curved sections with bending deflection and stretched flange are produced at *S* of 4.4 mm. For kerf depth *h* of 1 mm (Figure 6b), the material damage concentrates along with the kerf tips, and the shearing displacement *S* is reduced to 0.8 mm for damage initiation. With *S* from 0.8 mm to 1.1 mm, crack seams propagate from the top and bottom kerf edges vertically, and highly flat sections are produced at a shorter displacement of 3.5 mm.

**Figure 7:**
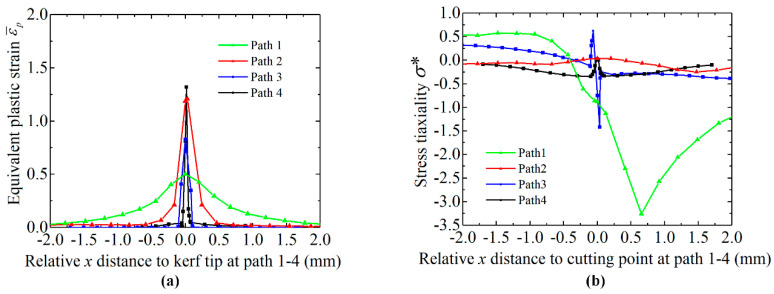
Equivalent plastic strain ${\bar{\varepsilon }}_{p}$ and stress triaxiality σ* along with path 14. (**a**) ${\bar{\varepsilon }}_{p}$. (**b**) σ*.

Figure 7 illustrates the distribution of equivalent plastic strain ${\bar{\varepsilon }}_{p}$ and stress triaxiality σ* along with paths 1–4; paths 1 and 3 are along with the top surface of the bar; paths 2 and 4 are along with the middle surface of the bar; x distance is zero at the cutting point or kerf tip. For the bar without kerf, the equivalent plastic strain ${\bar{\varepsilon }}_{p}$ gradually varies to a peak value of 0.50 at the cutting edge at the top surface (path 1); the corresponding stress triaxiality σ* varies from 0.54 to −3.26 and back to −1.71 (path 1). This reveals that the adjacent material of the cutting point has undergone plastic deformation by tension stress at the left side, and by compression stress at the right side. For the bar with kerf, the equivalent plastic strain varies as a sharp curve with the maximum value of 0.83 at the kerf tip (path 3), which shows that the plastic deformation only concentrates at the kerf zone. Compared to the bar without kerf, the equivalent plastic strain ${\bar{\varepsilon }}_{p}$ reaches a higher value of 1.21 at the middle surface kerf tip (path 4), and drops rapidly to 0 at both sides; the corresponding stress triaxiality varies around a smaller range of 0.32 to −0.38 and reaches 0.08 at the kerf tip (path 4), which reveals that shear stress plays a major role in the crack initiation for the bar with surface kerf.

**Figure 8:**
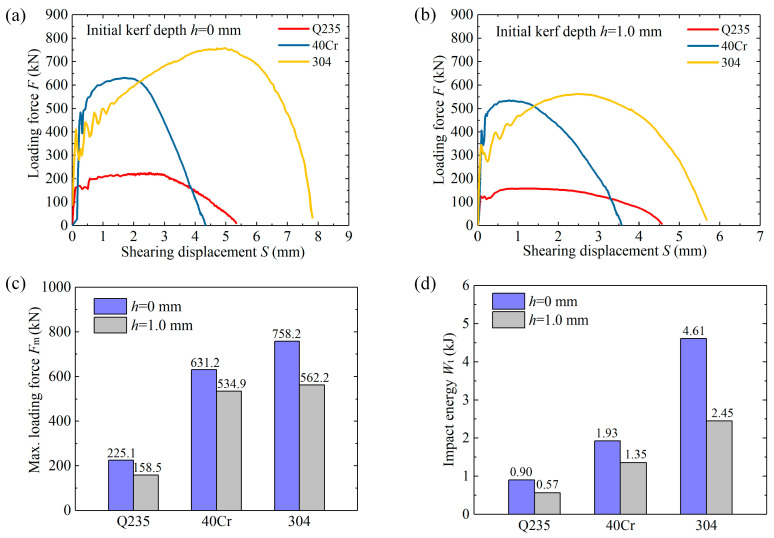
Loading force-displacement curves. (**a**) *F*-*S* curves for *h* of 0 mm. (**b**) *F*-*S* curves for *h* of 1.0 mm. (**c**) Max. loading force $F_{m}$. (**d**) Impact energy $W_{t}$.

Figure 8 compares the *F*-*S* curves and impact energy $W_{t}$ in the double-shearing process. These curves were already smoothed with the method of adjacent averaging to decrease the fluctuation due to high-speed load. For the three types of bar material, the Max. loading force $F_{m}$ decreases by 15.2%–29.6%, and impact energy $W_{t}$ reduces by 29.8%–46.9%. Taking the 40Cr bar as an example, Max. loading force $F_{m}$ decreases by 15.2% from 631.2 kN to 534.9 kN, and impact energy $W_{t}$ drops 29.8% from 1.93 kJ to 1.35 kJ.

Above all, the simulated fracture profiles are very consistent with the experimental results, which validates the credibility of the FE model. The fracture-start kerfs effectively inhibited the plastic deformation stage; the material damage concentrates along with the kerf tips with peak equivalent plastic strain subjected to shearing stress at a low-stress triaxiality. The three types of bar material present a good kerf-sensitivity performance at low stress triaxial; the 304 stainless steel shows preeminent ductility, strain-hardening, and fracture toughness due to additions such as nickel (Ni), chromium (Cr), and manganese (Mn). This method compensates for the drawbacks of high-speed cropping methods: it not only improves the blank quality, but also reduces the Max. loading force and impact energy, which is energy-saving and beneficial for the shearing die and equipment.

### 4.2. Macro-Fractography Analysis

#### 4.2.1. Influence of Fracture-Start Kerf on the Macro-Fractography

The section quality is evaluated by indexes of roundness error $e_{r}$, Max. bending deflection $e_{b}$, and flatness error $e_{f}$ with standard error. The $e_{r}$ and $e_{b}$ is both measured by a three-coordinate measuring system; the $e_{f}$ is measured by VHX-500 hyperdepth three-dimensional microscope.

**Figure 9:**
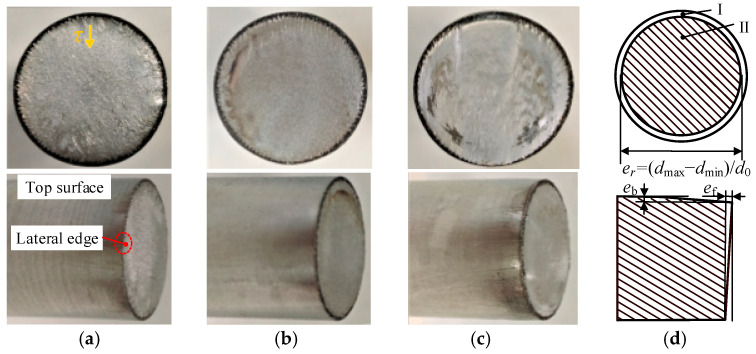
Macro-fractography for Test 1 with circumferential kerf. (**a**) Q235. (**b**) 40 Cr. (**c**) 304. (**d**) Evaluation indexes.

In test 1 with circumferential kerf (Figure 9), the macro-fractography typically shows highly flat fracture planes with laser affected zone at the outer edge. Besides, the top surface material does not suffer much bending deflection due to the stress concentration effect at the kerf tip. For the three types of bar material, the section quality of the 40Cr steel is prominent with a roundness error $e_{r}$ of 1.1%, Max. bending deflection $e_{b}$ of 0.4 mm, and flatness error $e_{f}$ of 0.3 mm.

**Figure 10:**
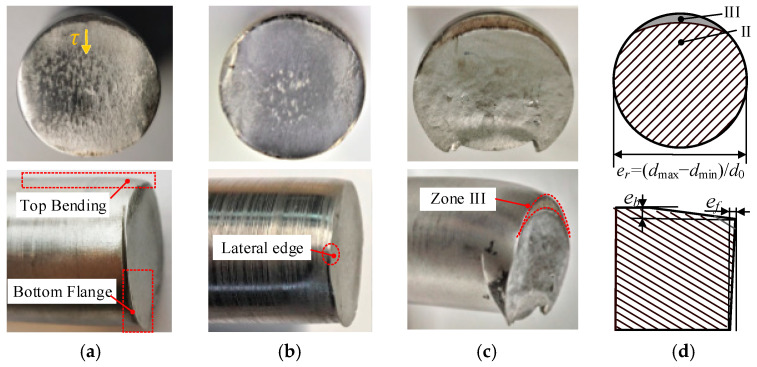
Macro-fractography for Test 2 without circumferential kerf. (**a**) Q235. (**b**) 40 Cr. (**c**) 304. (**d**) Evaluation indexes.

In contrast test without circumferential kerf (Figure 10), the fracture sections are slightly inward-curved, with bending deflection at the top surface, and stretched flange at the bottom edge. Typically, crescent-shaped shear lips occur at the top edge (zone III), which reveals that the local material is not only squeezed downward under radial compression stress, but also undergoes large axial compression stress due to the bending effect by the shear dies. At the bilateral edges, material accumulates and stretched downward into flanges at the bottom edges. The section quality is directly affected by the strength and plasticity of material: for low-carbon steels as 304 (0.06% C) and Q235 (0.21% C), it requires a severer accumulation of plastic deformation to give rise to failure.

**Figure 11:**
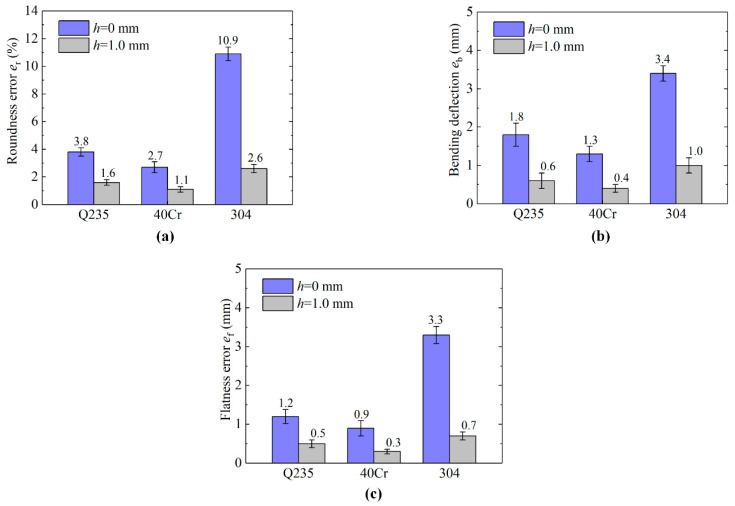
Section quality results of Test 1–2 for the three types of metal bars. (**a**) Roundness error $e_{r}$. (**b**) Max. bending deflection $e_{b}$. (**c**) Flatness error $e_{f}$.

In a comparison of test 1–2, higher precision section quality is obtained in the LAHSS test: the roundness error $e_{r}$ improves from 2.7%–10.9% to 1.1%–2.6%, the Max. bending deflection $e_{b}$ decrease from 1.3–3.4 mm to 0.4–1.0 mm, and the flatness error $e_{f}$ drops from 0.9–3.3 mm to 0.3–0.7 mm for the three types of materials (Figure 11). Taking 304 steel bars for example, the roundness error $e_{r}$ reduces by 76.1% from 10.9% to 2.6%, the Max. bending deflection $e_{b}$ reduces by 70.6% from 3.4 mm to 1.0 mm, and the flatness error $e_{f}$ drops by 84.8% from 3.3 mm to 0.7 mm.

#### 4.2.2. Influence of Axial Clearance on the Macro-Fractography

**Figure 12:**
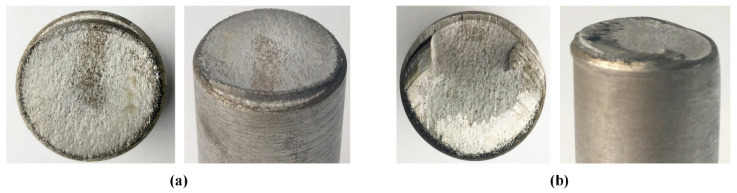
Macro-fractography for Test 3–4 with different axial clearance for 40Cr steels. (**a**) *C*_1_ = 1 mm. (**b**) *C*_1_ = 2 mm.

Test 3–4 shows that the section quality decreases with the increasing of the axial clearance *C*_1_ from 0.2 mm to 1 mm and 2 mm (Figure 12). The start-fracture location deviates from the top-surface kerf tip, and produces fracture planes with extrusion layers. It reveals that the material within the gap of shear dies experiences plastic deformation under bending, so that the crack initiation at the top-surface starts along with the blade edges instead of the kerf tip.

Above all, this LAHSS method effectively inhibits the plastic distortion and improves section quality with kerf depth of 1 mm, loading speed of 4.9 m/s, and axial clearance of 0.2 mm. It is feasible for soft Q235 steel, and high-strength 40Cr steel, with face-centered cubic (BCC) crystal structure, and hard-to-cut 304 steel with face-centered cubic (FCC) crystal structure. As for the light alloys, such as close-packed hexagonal (HCP) titanium alloys, and FCC aluminum alloys, further research will be done to investigate the feasibility and fracture behavior.

### 4.3. Micro-Fracture Mechanism Analysis

The micro-fractography of the laser-affected zone and main fracture plane, respectively, was taken by field-emission scanning electron microscope (Zeiss Gemini SEM 500) on the cross-section.

#### 4.3.1. Crack Initiation at Laser-Affected Zone I

**Figure 13:**
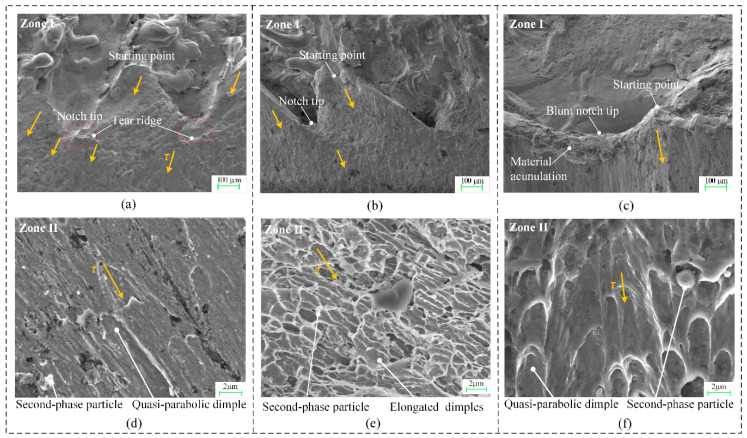
SEM fractography for the three types of bar material in Test 1. (**a**) Laser-affected zone I of Q235 bar. (**b**) Laser-affected zone I of 40Cr bar. (**c**) Laser-affected zone I of 304 bar. (**d**) Main fracture zone II of Q235 bar. (**e**) Main fracture zone II of 40Cr bar. (**f**) Main fracture zone II of 304 bar.

As shown in Figure 13a–c, the kerf bottom presents alternative V-shape micro-notches, which are continuously connected by laser-drilled micro-holes. The micro-notch tips become blunt to some extent with larger opening angle and apex radius, which is the most for the 304 stainless steel bars with material accumulation, and least for the 40Cr steel bars. As cracks usually start from the stress concentrators on the free surface, it can be seen that micro-cracks start from the transition pints of two adjacent micro-holes, propagate downward, and converge into tear ridges around the micro-notch tips. After that, larger cracks propagate from the tear ridges and eventually coalesce into a continuous fracture plane.

#### 4.3.2. Microvoid and Grain Distribution at the Main Fracture Zone II

As shown in Figure 13d–f, the fractography is covered by shallow quasi-parabolic dimples, revealing that the predominant fracture mechanism involves mode II microvoid coalescence. Moreover, second-phase particles are dispersed at the bottom of these dimples. Generally, second-phase particles or inclusions provide the nucleation sites for microvoids due to localized shear strain concentrations under overload.

**Figure 14:**
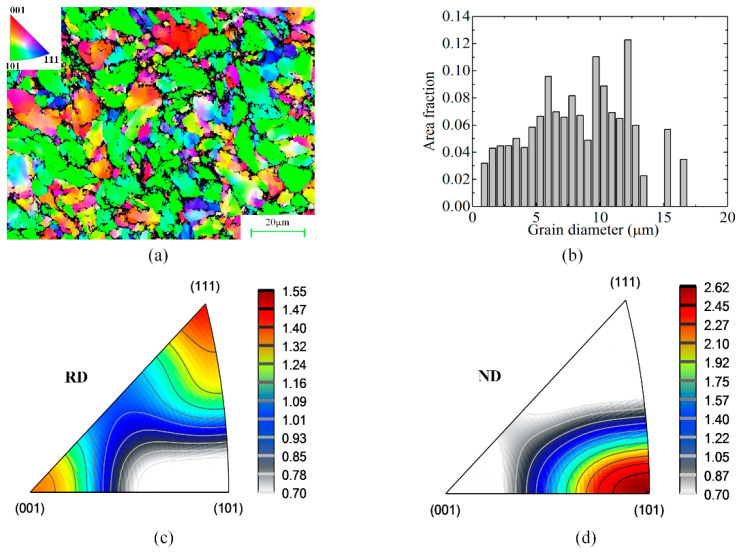
EBSD results: grain size distribution and inverse pole figures (IPFs) of 40Cr bar in Test 1. (**a**) EBSD crystal direction map. (**b**) Grain size distribution. (**c**) IPF of the rolling (cropping) direction. (**d**) IPF of the normal direction.

Among the three types of bar material, smaller and less elongated dimples are formed in 40Cr steel, which has a higher number density of grains with an average size of 11.9 μm measured in electron back scattering diffraction (EBSD) analysis (Figure 14a–b), revealing that the dislocation movement is better blocked due to the grain refinement and higher pinning effect of second-phase particles at the grain boundaries. The corresponding inverse pole figures (IPFs) of the 40Cr steel are as shown in Figure 14c–d. As well known that the slip systems in body-centered cubic metals generally consist of slip planes of (110) and (112) with slip direction of <111>, and it can be seen a certain degree of (110) <111> and (110) <001> texture are present with maximum statistic value of 2.62, which shows that the plastic deformation is not so severe and strongly textured in this radially constrained LAHSS process.

## 5. Conclusions

This study presents a LAHSS method for high-quality cropping of metal bars. The fracture behavior, macro-fractography, and micro-fracture mechanism of three types of bar material were comparatively investigated in this high-speed shearing process. The main conclusions are as follows:

1. The FE simulation results show that by introducing initial kerf of 1.0 mm, the material damage concentrates along with the kerf tips with peak equivalent plastic strain, and the corresponding stress triaxiality drops to almost zero at the kerf tip, which reveals that the material is subjected to pure shearing at kerf tip, and under compression at both sides; shear strain plays a major role in the fracture process. Moreover, the Max. shearing force is reduced by 15.2%–29.6%, and the impact energy is decreased by 29.8%–46.9% for the three types of bar material.
2. The experimental results showed that this LAHSS method effectively inhibited the plastic distortion and improves section quality: the roundness error improves from 2.7%–10.9% to 1.1%–2.6%, Max. bending deflection decrease from 1.3–3.4 mm to 0.4–1.0 mm, and flatness error drops from 0.9–3.3 mm to 0.3–0.7 mm for the three types of bar material. The section quality decreases with the increasing of the axial clearance from 0.2 mm to 1 mm and 2 mm.
3. The fractographic analysis reveals that the crack initiation is related to alternative V-shape micro-notches at the laser-affected zone; the predominant fracture mechanism involves mode II microvoid coalescence at the main fracture plane; smaller and less elongated dimples were formed in 40Cr steels due to higher number density of grains and pinning effect of second-phase particles compared to Q235 and 304 steel bars.

## Figures and Tables

**Table 1:** The processing parameters in the shearing tests.

Test No.	*h* (mm)	*E* (kJ)	$v_{1}\;(m/s)$	$C_{1}\;(\mathrm{mm})$	$C_{2}\;(\mathrm{mm})$
1	1.0	15.6	4.9	0.2	0.2
2	0.0	15.6	4.9	0.2	0.2
3	1.0	15.6	4.9	1.0	0.2
4	1.0	15.6	4.9	2.0	0.2

**Table 2:** The Johnson–Cook plasticity and damage parameters of the bar materials.

Materials	Johnson–Cook Plasticity Parameters						Damage Parameters				
	*A*(Mpa)	*B*(MPa)	*n*	*m*	*C*	${\dot{\varepsilon }}_{0}\;$	*d_1_*	*d_2_*	*d_3_*	*d_4_*	*d_5_*
Q235 [27]	213	53	0.345	0.81	0.055	0.004	0.05	3.44	−2.12	0.002	0.61
40Cr [28]	792	510	0.26	1.03	0.014	1	0.1	0.76	–1.57	0.005	–0.84
304 [27]	310	1000	0.65	1	0.07	0.1	0.53	0.5	−6.8	−0.014	0.0

## Nomenclature

2α	Opening angle of the kerf	
A,B,c,n,m	Material parameters of the Johnson-Cook flow stress model	

$C_{1}$	Axial clearance between the movable and the fixed shear dies	

$C_{2}$	Radial clearance between the shear dies and the metal bar	

$d_{1},d_{2},d_{3},d_{4},d_{5}$	Material parameters of the Johnson-Cook fracture model	

$d_{0},d_{\max},d_{\min}$	Original, maximum, and minimum diameters of the fracture section	

dt	Increment of time	
D	Johnson-Cook normalized cumulative damage value	

$\sigma_{1},\sigma_{2},\sigma_{3}$	Three principle stresses	
$\sigma_{m}$	Average of the three principal stresses	
$\bar{\sigma }$	Von Mises equivalent stress	
σ*	Stress triaxiality	
${\dot{\varepsilon }}_{0}$	Reference strain rate	
${\dot{\varepsilon }}_{1},{\dot{\varepsilon }}_{2},{\dot{\varepsilon }}_{3}$	Three principle strain rates	
${\dot{\bar{\varepsilon }}}_{p}$	Equivalent plastic strain rate	
${\bar{\varepsilon }}_{p}$	Equivalent plastic strain	
$\Delta {\bar{\varepsilon }}_{p}^{}$	Increment of equivalent plastic strain	
${\bar{\varepsilon }}_{f}$	Equivalent fracture strain	
$e_{b}$	Maximum bending deflection of blank	
$e_{f}$	Flatness error of blank	
$e_{r}$	Roundness error of blank	
E	Kinetic energy of hammer	
F	Loading force by hammer	
$F_{m}$	Maximum loading force	
S	Shearing displacement	
η	Efficiency in the impact stroke	
h	Kerf depth	
k	Gas adiabatic constant	
L	Cropping length of blank	
$m_{1}$	Hammer mass	
$m_{2}$	Total mass of the floating block and the movable shear die	
$p_{1}$	Gas pressure before expansion	
$p_{2}$	Gas pressure after expansion	
r	Curvature radius of the kerf tip	
T	Workpiece temperature	
$T_{m}$	Melting temperature	
$T_{r}$	Reference temperature	
$v_{1}$	Loading speed of the hammer	
$V_{1}$	Gas volume before expansion	
$V_{2}$	Gas volume after expansion	
w	Kerf width	
$W_{g}$	Work done by hammer gravity	
$W_{p}$	Work done by gas expansion	
$W_{t}$	Impact energy in shearing process	
$z_{1}$	Working stroke of hammer	
